# Elements Involved In Promoting Eosinophilic Gastrointestinal Disorders

**DOI:** 10.4172/2157-7412.1000265

**Published:** 2015-08-07

**Authors:** Anshi Shukla, Akanksha Mishra, Sathisha Upparahalli Venkateshaiah, Murli Manohar, Chandrashekara Puthanapura Mahadevappa, Anil Mishra

**Affiliations:** Department of Medicine, Section of Pulmonary Diseases, Tulane Eosinophilic Disorder Center, 1430 Tulane Avenue, New Orleans, LA 70112

**Keywords:** Eosinophils, EoE, EGE, EGID, Food allergy, Interleukin, iNKT cells

## Abstract

Eosinophilic gastrointestinal disorders (EGID) are food allergen-induced allergic gastrointestinal disorders, characterized by accumulation of highly induced eosinophils in different segments of gastrointestinal tract along with eosinophil microabssess and extracellular eosinophilic granules in the epithelial layer. EGID are both IgE- and cell-mediated group of diseases that include eosinophilic esophagitis (EoE), eosinophilic gastritis (EG), eosinophilic gastroenteritis (EGE) and eosinophilic colitis (EC). Despite the increased incidences and considerable progress made in understanding EGID pathogenesis. The mechanism is still not well understood. It has been shown that IL-4, IL-5, IL-13, IL-15, IL-18, eotaxin-1, eotaxin-2 and eotaxin-3 play a critical role in EGID pathogenesis. Currently, the only criterion for diagnosing EoE, EGE and EC are repetitive endoscopic and histopathological evaluation of biopsies along with other clinical characteristics/manifestations. Antigen elimination and corticosteroid therapies are the most effective therapies currently in practice for the treatment of EGID. The cytokines (anti-IL-5 and anti-IL-13) therapy trials were not very successful in case of EoE. Most recently, a clinical trial using anti-IL-13 reported only 60% reduced esophageal eosinophilia without achieving primary endpoint. This clinical finding is not surprising and is in accordance with our earlier report indicating that IL-13 is not critical in the initiation of EoE. Notably, EGID still has no reliable noninvasive diagnostic biomarkers. Hence, there is a great necessity to identify novel noninvasive diagnostic biomarkers that can easily diagnose EGID and provide an effective therapy. Now, the attention is required to target cell types like iNKT cells that produce eosinophil active cytokines and is found induced in the pathogenesis of both experimental and human EoE. iNKT cell neutralization is shown to protect allergen-induced EoE in experimental model. In this review, we have discussed the key elements that are critical in the disease initiation, progression, pathogenesis and important for future diagnostic and therapeutic interventions for EGID.

## Introduction

**Figure 1:**
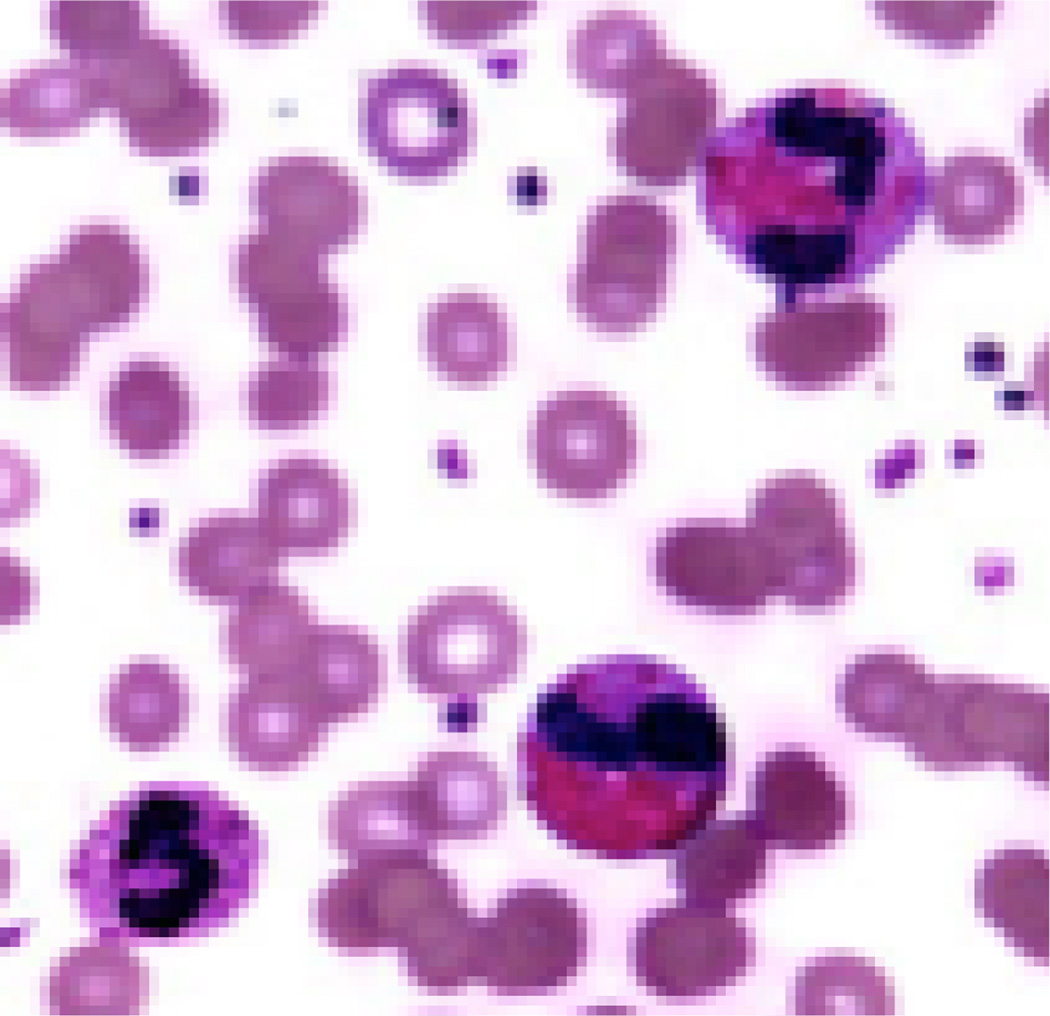
Blood Eosinophils. The blood eosinophils morphology is shown by microscopic analysis of Gimmsa stained blood smear slide. (Original magnification ×400).

Eosinophils are an important subtype of blood leukocytes and are differentiated from multipotent hematopoietic stem cells in the bone marrow from myeloid lineage myeloblasts [1,2]. These eosinophils are multifunctional leukocytes that are involved in vivid innate and adaptive immune responses [2–4]. Eosinophils home into the gastrointestinal tract in prenatal period, independent to bacterial flora [5]. Baseline eosinophil number varies depending upon the geographic condition and seasonal variations [6–8]. Eosinophils are reported to initiate inflammatory and adaptive responses because of their interactions with antigen presenting cells and T cells, along with their propensity to synthesize numerous cytokines and a number of mediators. They play a significant role in host defense, regulation of the immune system and in the eradication of parasitic infection [9]. Eosinophils also have a significant role in healing and organogenesis before birth [10]. Increased level of eosinophilic accumulation in tissue or blood (Figure 1) with marked degranulation is reported in a number of inflammatory diseases; like asthma, eosinophilic dermatitis, gastroesophageal reflux, celiac disease, inflammatory bowel disease, allergic colitis, food allergy and parasitic infections, In normal conditions eosinophils are found in each segment of the GI tract from the stomach to the colon in the lamina propria except the esophagus, Peyer’s patches, or intra-epithelial locations [4,5,11–20]. Further, they are known to have diverse roles in the gastrointestinal tract, which includes excretion of intestinal parasites. Although, it is believed that peristalsis is the major cause of the excretion of intestinal parasites, despite this role of eosinophil in parasite eradication is not ruled out in healthy state, and their stimulation promotes the pathogenesis of various allergic gastrointestinal disorders like drug reactions, food allergy, parasitic infection, hypereosinophilic syndromes, allergic colitis, gastroesophageal reflux disease, inflammatory bowel disease. Interleukin (IL)-5 is a well-established differentiation, growth and survival factor for eosinophils; however, eosinophil lineage commitment, differentiation, effector functions, and their roles in various diseases are under renewed scrutiny [21–25]. Yet, it is not clearly understood whether a different subpopulations of eosinophils exists in health and disease. The recruitment of eosinophils in the tissues of IL-5 gene-deficient mice and failed therapeutic trials with humanized anti-IL-5 monoclonal antibodies in asthma and other gastrointestinal disorders, indicate that eosinophils may have different subsets [5,26]. It may be possible that IL-5 independent eosinophil subset might exist in a health and disease state and has to be explored. We earlier reported that baseline eosinophils exist in IL-5 gene-deficient mice; therefore, it is rationale to explore the characteristics of the eosinophil population that exist in IL-5-independent environment [27]. Hence, the researchers and biologist involved in studying eosinophil biology need to explore this possibility and investigate the factors that are responsible for the differentiation and survival of eosinophils in IL-5 gene-deficient mice. The presence of IL-5 independent eosinophil subset may provide a new understanding on eosinophil biology in health and disease.

### Eosinophils trafficking and homing occurs by its specific chemoattractant, receptors and adhesion molecules

Eosinophil accumulation often occurs in the absence of other inflammatory cell infiltration in the tissues; therefore, a number of studies were focused on identifying eosinophil specific chemoattractants [28]. Eosinophils respond to a variety of chemoattractants including eotaxin-1, -2, -3, RANTES, MIP-1α, MCP-2, -3, -4, and lipid mediators like PAF, LTB4, LTC4. Eotaxin-1 was first identified to selectively attract eosinophils when injected into the skin of naive guinea pigs and established as a selective eosinophil chemoattractant [29–31]. Further, a molecular approach was utilized to characterize eotaxin-1 gene and cDNA in guinea pig, murine and human eotaxin-1 [32–34]. Eotaxin-1 was a unique chemokine most homologous to the MCP chemokines [35]. In addition, eotaxin has also been shown to be a potent activator of eosinophils [36]. Subsequently, the other eotaxin families of cytokines were also identified and shown that the activity of sub groups is largely tissue specific, example; eotaxin-1 is a specific chemoattractant for eosinophil in the gastrointestinal tract and eotaxin-2 mostly active in the lung. Further, eotaxin-3 is specifically expressed in the lung and gastrointestinal segments in human tissues. The specificity of eotaxin for eosinophils is the result of the exclusive signaling of eotaxin-1, eotaxin-2 and eotaxin-3 through its receptor, CCR3, which is expressed predominantly on human and mouse eosinophils [30–32]. Eosinophil-selective chemokine, eotaxin-2, and eotaxin-3 have 30% homology to eotaxin-1 and were discovered in last decade by employing empirical genomic methods [37–40]. Based on C-C motif they are also termed as CCl-11, CCl-24 and CCL-26. Interestingly, eosinophils also express several chemokine receptors, but CCR3 is expressed at the highest level [37,38]. Significantly lower levels of CCR1 (~30,000 receptors/cell) are expressed on eosinophils from most healthy individuals. CCR3 appears to function as the major eotaxin specific receptor for eosinohils. In addition, eosinophils also respond to MIP-1α, RANTES, MCP-2, MCP-3 and MCP-4 [41]. Eosinophil express numerous adhesion molecules including α4β1 (also known as VLA-4), β7 integrins, ICAM, and VCAM [42,43]. These adhesion molecules mediate uneven degrees of eosinophil homing and activation, but β7 seems to be most critical in facilitating eosinophil homing into the GI tract [43–45]. The receptor for β7 integrin, MAdCAM-1, contains three immunoglobulin domains and one mucin-like domain and is expressed by the high endothelial venules of lymph nodes and by flat endothelium in the lamina propria of intestine [42]. In the GI tract, several studies have shown that eosinophils are present in the lamina propria along with resident neutrophils and macrophages [43,46]. Similar to the distinct properties and functions of other GI immune cells, it is anticipated that GI eosinophils will have unique properties.

### Eosinophils are the source of a number of proinflamatory and profibrotic proteins

Activated eosinophils generate a wide range of inflammatory cytokines including IL-1, IL-3, IL-4, IL-5, IL-13, IL-18 GM-CSF, TGF-α/β, TNF-α/β indicating that they have the potential to regulate various features of the immune response [47,48]. Eosinophils degranulate via two different mechanism, they are commonly observed to undergo fragmentary degranulation, whereby the granular content is released from intracellular granules and undergo cytolysis, whereby the cell membrane ruptures, causing the release of free eosinophil granules [5,49–52]. Eosinophil has four major granules that contain certain pro-inflammatory properties. MBP, EPO, and ECP have cytotoxic effects on epithelium, in concentrations similar to those found in biological fluids from patients with eosinophilia [53]. Additionally, ECP and EDN belong to the ribonuclease super-family and possess anti-viral and ribonuclease activity [54,55]. Add Major basic Protein (MBP) directly increases smooth muscle reactivity by causing dysfunction of vagal muscarinic M2 receptors [56]. Major basic protein also triggers degranulation of mast cells and basophils. Further damage is caused by toxic hydrogen peroxide and halide acids generated by EPO and by superoxide generated by the respiratory burst oxidase enzyme pathway in eosinophils [11,57]. Eosinophils also generate large amounts of the cysteinyl leukotriene, leukotriene C4 that metabolizes leukotriene D4 and leukotriene E4 [56]. These lipid mediators increase vascular permeability and mucous secretion, and are potent stimulators of smooth muscle contraction [48]. Eosinophil stimulates inflammatory cell recruitment and triggers the secretion of fibrogenic mediators resulting in tissue remodeling, disruption of epithelial integrity by affecting the fibroblasts, smooth muscles, and cell-adhesion molecules [11,13,58–60].

### Eosinophils have a significant role in promoting allergic diseases

The occurrence of environmental and food allergies is becoming significantly higher throughout the world. Allergic diseases such as allergic dermatitis (AD), allergic rhinitis (AR), allergic asthma (AA), and allergic gastrointestinal diseases (AGD) are commonly related with food and aeroallergen-induced allergies. Theses allergic diseases affect hundreds of millions of individuals globally. Incidence of allergic diseases creates a major global health concern [61,62]. Evidences indicate that food allergy is of significant concern among the increase occurrences of allergic diseases. Food allergy is a lack of oral tolerance to food that is ingested [6]. It is an immune-based disease that has posed a serious public health apprehension worldwide affecting approximately 5% of children under 5 years and 4% of teens and adults, and is showing enhanced incidences. The most common type of food allergy that is found in adults and older children, largely involve an immune response to the ingested food allergens (oral allergy syndrome) and inhalant allergen (pollen-food syndrome) that consequently inflames a secondary immune response to a cross-reactive allergen which is being ingested [62]. Further, the symptoms of food-related allergic diseases could be mild to severe, and have emerged as a leading cause of anaphylactic reactions resulting in approximately 30000 emergency department visits, culminating in about 150–200 deaths each year in the United States. It is expected that antigen presenting cells which include epithelial cells, dendritic cells, and B cells present the food antigens to naïve T cells that further leads to the formation of Th2 type effector T cell specific to food antigens. These effector cells then release a collection of Th2 cytokines (IL-4, IL-5, IL-9, IL-13, IL-15 and IL-18) that are organized in the intestinal immune system for prompting food allergy or anaphylactic immune responses upon successive exposure to the antigen [51,63]. Food allergy generally results from food proteins that are stable to digestion, and is usually found in infants or children where the immune system is undeveloped and also developed due to sensitization to certain proteins that are confronted in the GI tract and are vulnerable to enzymatic degradation leading to IgE production. These IgE identify homologous epitopes on food proteins generating allergic responses. Experimental systems have demonstrated that inflammatory responses in allergic diseases are often biphasic, but this has not been well uncovered in the upper and lower GI tract. The early phase has been shown to directly involve IgE mast cell-associated release of histamine, prostaglandin-D2, and leukotrienes [64,65]. Th2 cells are thought to induce late phase reactions through the exudation of a number of cytokines. In particular, IL-4, IL-13, and IL-18 are produced at elevated levels in allergic tissue and are thought to be central regulators of many of the characteristic features of the disease [66]. IL-4 promotes Th2 cell differentiation, IgE induction, tissue eosinophilia, and, in the case of asthma, morphological changes to the respiratory epithelium [67,68]. IL-13 is considered to be more of an effector cytokine in disease pathogenesis compared to IL-4, as indicated by the finding that soluble IL-13 receptor blocks many crucial properties of the experimental disease [60,69,70]. Although IL-4 and IL-13 have only 25% homology, they share receptor components and have many coinciding functional characteristics that underlie their role as critical regulators of many of the trademark features of allergic disease [27,71]. Studies in signal transducer and activator of transcription (STAT)6 gene-deficient mice have revealed that IL-13 signaling utilizes the JAK-STAT pathway, precisely STAT6, and that mice with targeted removal of STAT6 gene have diminished development of Th2-associated reactions in the GI tract following parasitic infection [70,72,73]. The patients exhibiting food allergy associated gastrointestinal symptoms like vomiting, diarrhea and bloody stool are displayed after intake of specific foods. These patients are categorized under a subgroup of food allergy associated gastrointestinal disorders [74–76]. A number of cells that include dendritic cells, macrophages and T cells are involved in the processing and sampling of these food antigens in the GI mucosa [77,78]. In normal condition the immune system of the gastrointestinal mucosa evades immune reactivity to harmless foreign antigens and this is called “oral tolerance” [16,17]. The GI allergy could be either “cell-mediated non-IgE-mediated” or “combined IgE- and cell-mediated” and it includes several different clinical characteristics that include food protein-induced enterocolitis syndrome (FPIES), food protein-induced enteropathy, food protein-induced proctocolitis (FPIP), and eosinophilic gastrointestinal disorders (EGID). The gastrointestinal (GI) tract absorbs only about 2% of ingested food antigens and a number of cells including intestinal epithelial cells, dendritic cells, B cells, and T cells are involved in processing food antigens in the GI mucosa [77–80]. It is postulated that antigen presenting cells (epithelial cells, dendritic cells, and B cells) present luminal food antigens to naïve T cells leading to the progression of a food antigen-specific Th2 type effector T cell populations. These effector cells, through exposure to an array of Th2 cytokines (IL-4, IL-5, IL-9, IL-13, IL-15 and IL-18) in the intestinal immune system, are primed for food allergy or anaphylactic reactions upon subsequent antigen exposure [51,63]. Eosinophils, mast cells and basophils along with IgE have an important role in inducing anaphylactic reactions. IL-13 is considered to be more of an effector cytokine in pathogenesis compared to IL-4, as suggested by the finding that a soluble IL-13 receptor blocks many essential qualities of the experimental disease [60,69,70]. Most recently, we showed a critical role of food allergen-induced IL-18 and invariant natural killer T cells in human and experimental gastrointestinal allergic disease [3,13,81].

### Eosinophil associated gastrointestinal diseases

A large number of eosinophil accumulations in the segments of gastrointestinal tract promote a number of GI disorders that are commonly referred as eosinophilic gastrointestinal disorders (EGID). However, EGID also includes the presence of elevated mast cells and basophils in the tissues. EGID are combined IgE- and cell-mediated group of diseases that include eosinophilic esophagitis (EoE), eosinophilic gastritis (EG), eosinophilic gastroenteritis (EGE) and eosinophilic colitis (EC). They are characterized by eosinophilic infiltration in the different section of gastrointestinal (GI) tract with varying GI symptoms and are reported to occur in the absence of known causes of eosinophilia like parasitic infection, neoplasm, vasculitis and food allergy [3,82].

## Eosinophilic esophagitis

**Figure 2:**
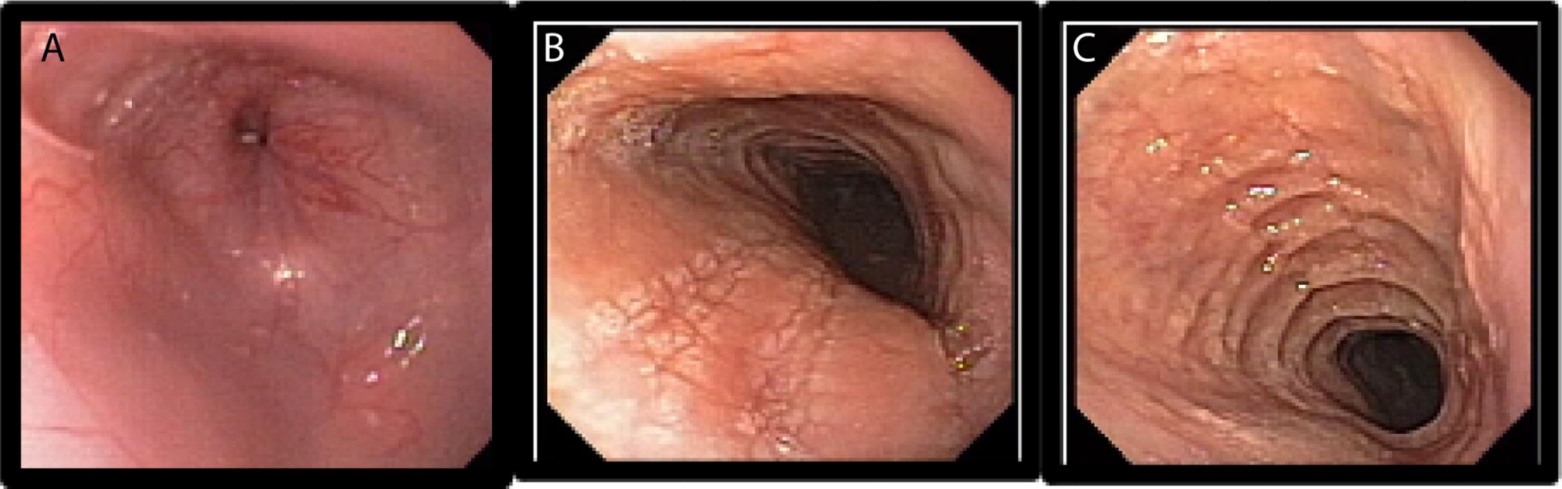
Endoscopic evaluation of the EoE patient esophagus. Esophageal furrows and ring formation in EoE patients. A representative photomicrograph of a normal esophagus (A), and the development of esophageal furrows (B) and rings (C) are shown in EoE patients.

**Figure 3:**
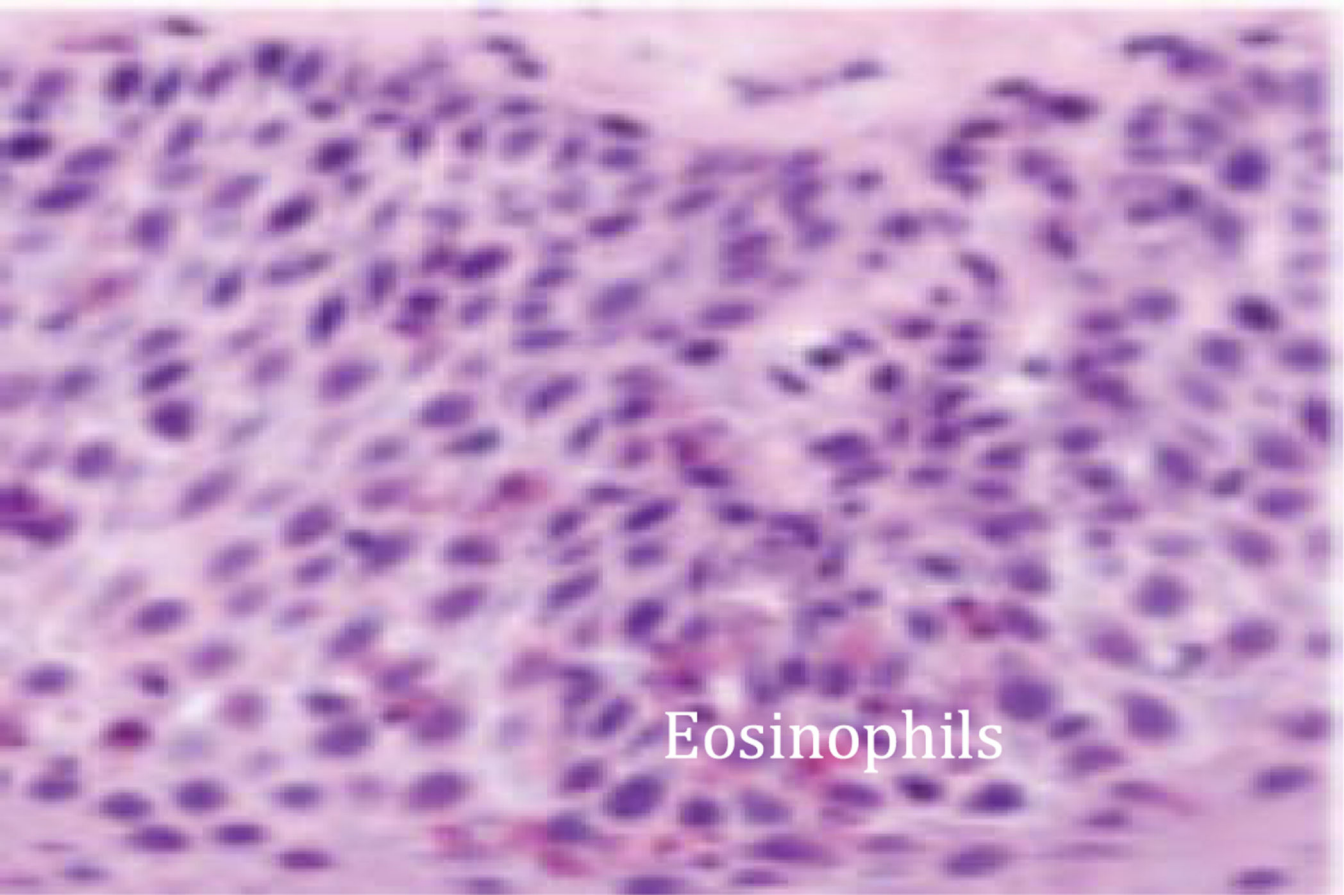
Eosinophils accumulation in esophageal mucosa. Induced levels of eosinophils accumulated in the esophageal mucosa of EoE patient is shown in the esophageal biopsy by staining the biopsy tissue section with H&E (Original magnification ×400).

Eosinophilic esophagitis (EoE, earlier also referred as EE) is one of the more common EGID conditions along with eosinophilic gastroenteritis (EGE). In recent years, there has been major progress in understanding the involvement of eosinophil and mast cell accumulation esophageal remodeling and functional esophageal abnormalities; however, the mode of EoE induction, its progression, diagnosis and it management are currently not well proven [11,13,58,59,83–85]. EoE was first described in 1978 and clinically recognized in 1994 further it was well accepted with distinct characteristics and nomenclature in 2007, and was recently updated in 2011 [86–89]. It is defined as a chronic immune/antigen mediated esophageal inflammatory disorder that involves inflammation associated with predominance of eosinophil leading to esophageal dysfunction [58]. It has been observed to be an increasingly important cause of upper gastrointestinal morbidity in adults as well as children’s over the past 2 decades. While EoE is characterized by infiltration of large numbers of eosinophils (≥ 15 eosinophils/high-powered field) in the epithelia lining of the mucosa of esophageal patients biopsies, lack of response to 8-week proton-pump inhibitor (PPI) trial and to acid-suppressive medication [5,20,87–89]. Esophagus is normally devoid of, eosinophils in individuals as well as in normal mice at baseline in healthy state [5,20]. EoE responds well to removal of dietary antigens and anti-inflammatory steroidal medications. Clinical symptoms of EoE vary with age, but are characterized by esophageal dysfunction that involves abdominal pain, dysphagia, and episodes of food impactions [90]. Children affected with EoE are typically observed to be incapable to thrive, with recurrent vomiting, feeding difficulties and heartburn [87,91]. Researchers have also identified specific endoscopic features that are associated with EoE include furrows, rings (Figure 2B, C) and stricture formation, narrowed esophagus, crepe-paper mucosa, linear furrows, white plaques, and histological findings like eosinophil degranulation, spongiosis, and subepithelial fibrosis [85,87,91–93]. Further, the histological investigation of EoE patients esophageal biopsies also demonstrate cellular infiltrates including eosinophils, (Figure 3) mast cells and their degranulated products, basophils and CD3+, CD4+, CD8+ T-cells and also invariant natural killer (iNK) T cells and mainly Th2 type inflammatory immune responses [59,81,94–100]. The Th2 response on being activated recruit and activate cell mediated non-IgE, type IV hypersensitivity. These cells and mediators lead to tissue fibrosis, where in esophageal epithelial cells provide a hospitable environment for the initiation of the inflammatory processes in EoE.

## Eosinophilic gastroenteritis (EGE)

**Figure 4:**
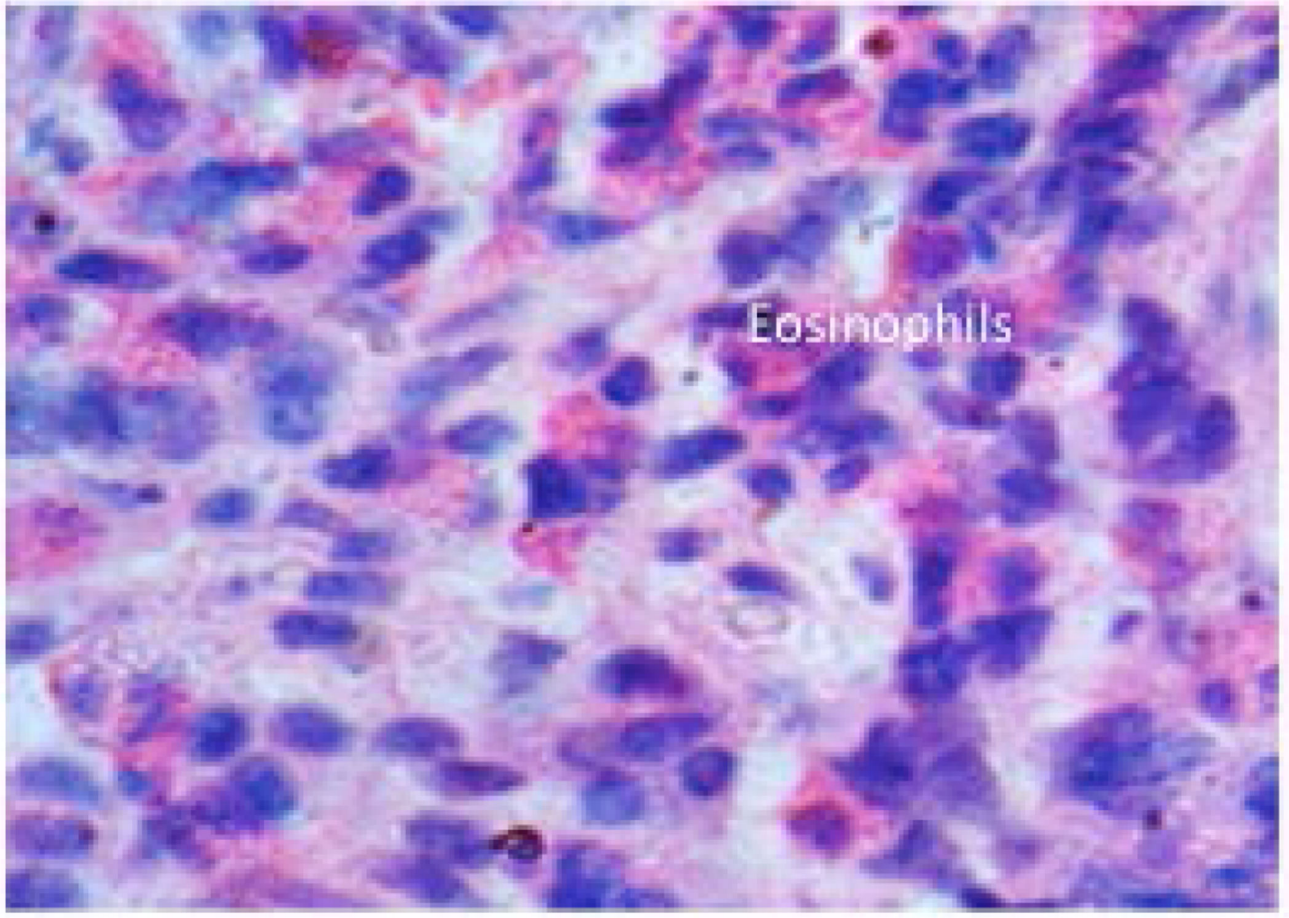
Eosinophils accumulation in Duodenum. Induced levels of eosinophils accumulated in the duodenal mucosa of EGE patient is shown by staining the tissue biopsy section with H&E (Original magnification ×400).

Eosinophilic gastritis, enteritis, and gastroenteritis are clinically similar disorders and are characterized by the selective infiltration of eosinophils in the stomach, small intestine and large intestine [101]. EGE is commonly described into three distinct type, e.g., mucosal, muscular and subserosal [102]. Eosinophil accumulation in the stomach occurs due to the parasitic and bacterial infections (including Helicobacter pylori), periarteritis, allergic vacuities, scleroderma, drug injury, and food hypersensivity; EGE is characterized as primary or secondary [103]. Primary EGE is associated with inherited connective tissue disorders and secondary could be due to a more generalized eosinophilic gastrointestinal disease caused by drug or food allergy. In the experimental model of eosinophil-associated gastrointestinal dysfunction, we showed a strong role of the chemokine eotaxin-1 [12,15,82]. In addition to eosinophils (Figure 4), mast cells are also increased in EGE, and a murine model of oral allergen-induced diarrhea has demonstrated a critical role of mast cells in the pathogenesis of this specific cardinal feature (allergic diarrhea) of EGE [104]. Food allergy has also been suggested in promoting eosinophilic gastroenteritis in the patients [105]. Approximately, 50% of patients with the mucosal form had a history of food allergy or intolerance [106]. Increased total IgE and food-specific IgE levels have been detected in the majority of patients. A majority of patients have positive skin test in responses to a variety of food antigens but do not show typical anaphylactic reactions, which is consistent with a delayed-type of food hypersensitivity syndrome in EGE. However, in some cases such as erosive gastritis and enteritis with prominent eosinophilia are also characterized by negative skin test responses and absence of specific IgE. Positive skin prick test may only be found in half of the patients tested due to non-IgE mediated food allergies; therefore, the clinical utility of the positive skin test results remain controversial [107]. In clinical studies increased secretion of IL-4, IL-5 and IL-13 by peripheral blood T cells has been reported in patients with eosinophilic gastroenteritis [105,108]. Notably, till date no standards for the diagnosis of eosinophilic gastritis or gastroenteritis exist, but a few findings support the diagnosis [109]. For example, the presence of increased eosinophils in biopsy specimens from the gastrointestinal tract wall, the infiltration of eosinophils within intestinal crypts and gastric glands, the lack of involvement of other organs, and the exclusion of other causes of eosinophilia (eg, infections and IBD) are supportive of eosinophilic gastroenteritis. Histologic analysis of the small bowel from patients with these disorders reveals that extracellular deposition of eosinophil granule constituents, and indeed, extracellular MBP and ECP are immunohistochemically detectable at increased levels in the biopsies of stomach and duodenum [53,101,106,110].

## Eosinophilic colitis (EC)

**Figure 5:**
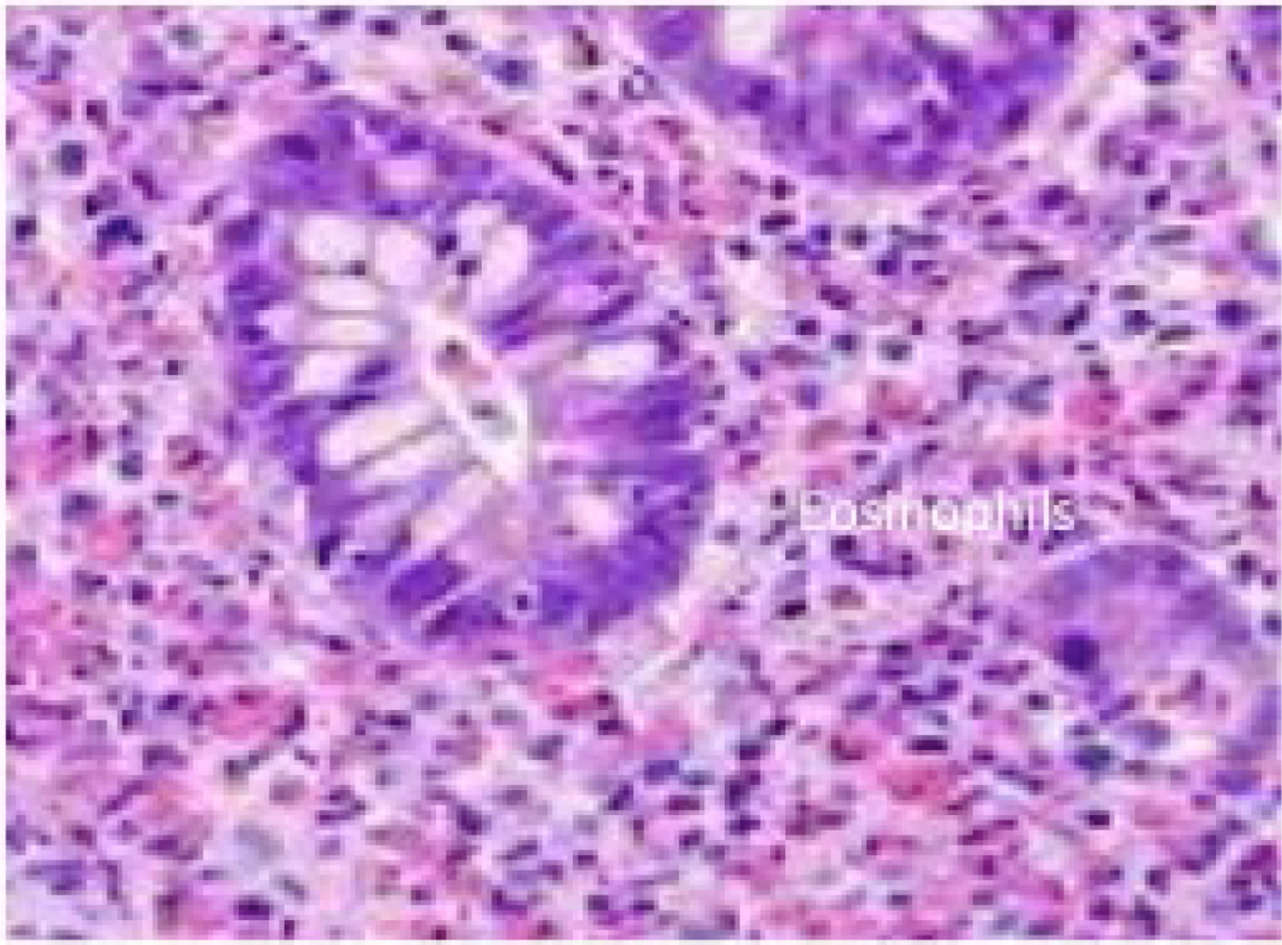
Eosinophils accumulation in Colon. Induced levels of eosinophil accumulated in the colon mucosa of EC patient is shown by staining the tissue biopsy section with H&E (Original magnification ×400).

Eosinophils accumulate in the colons of patients with a variety of disorders, including eosinophilic gastroenteritis and allergic colitis [2,5,111–114]. Allergic colitis is the most common cause of bloody stools. Eosinophilic colitis is usually a non-IgE–associated disease [115,116]. Some studies point to a T lymphocyte–mediated process, but the exact immunologic mechanisms responsible for this condition has not yet been identified [117]. It has been shown that oral antigen–induced diarrhea is associated with colonic inflammation of mast cells in experimental mouse model of colitis and might be an early expression of protein-induced enteropathy or protein-induced enterocolitis syndrome [18]. Food allergy is most frequently implicated in allergic colitis. Eosinophilic colitis, on endoscopic examination shows patchy erythema, loss of vascularity, and lymphonodular hyperplasia that are mostly localized to the rectum but might extend to the entire colon [18,117–120]. Histologic examinations often reveal focal aggregates of intraepithelial eosinophils (Figure 5), and also eosinophils in lamina propria, crypt epithelium, muscularis mucosa and, occasionally, the presence of multinucleated giant cells in the submucosa. No single test is the gold standard for diagnosis, but peripheral blood eosinophilia or eosinophils in the stool are suggestive of eosinophilic colitis [18]. Treatment of eosinophilic colitis in older individuals usually requires medical management because IgE-associated triggers are rarely identified. Anti-inflammatory drugs, including aminosalicylates and glucocorticoids (systemic or topical steroids) are commonly used to treat eosinophilic colitis. The natural history has not been well documented, and allergic colitis is considered to be a chronic waxing and waning disorder. Most infants with allergic colitis show symptom after 12 months of life. Eosinophilic colitis can often be a manifestation of other primary disease processes; therefore, routine surveillance of regular upper and lower gastrointestinal endoscopy is recommended and thus strengthening the need for further characterization of eosinophilic and allergic colitis.

## Treatment strategies of EGID

EGID, primarily involves food allergy; therefore, dietary treatment is a most common therapy for EGID. These dietary management approaches involve elimination or avoidance of specific foods from the patient’s diet base on food allergy testing results along with results from skin prick, atopy patch, and/or RAST testing. However, the food-specific IgE and skin prick can be used to identify the specific food allergens in EoE but alone are insufficient for the diagnosis. The most common foods that are eliminated or avoided include milk, peanuts, wheat, soy, egg, treenuts, corn, chicken, and beef. Further cow’s milk is observed as the most common food that triggers EGID. Removal of these foods in children with EoE was found to provide an 88% resolution to the disease. Further an elemental or hypoallergenic formula diet is also used as a dietary therapy that is the strictest and most effective form of dietary treatment. This includes intake of an elemental formula (including Neocate, Elecare, or Pur Amino) that comprises of amino acids that constitute the building blocks for proteins synthesis. This diet is devoid of any form of milk or soy proteins and is quite well tolerated and dose not elicits any allergic reaction. Reports suggest children with EoE on elemental diets show complete disease resolution in up to 95% cases. An antigen elimination approach in sensitized individuals (e.g., aeroallergen avoidance and food elimination diet) is typically unsatisfactory or practically difficult (when patients are sensitized to many allergens), likely because the current allergen sensitization tests (skin prick and patch tests, as well as antigen-specific plasma IgE levels) are not optimal for detecting sensitization. A diet consisting exclusively of an elemental (amino acid based) formula frequently improves symptoms and normalizes esophageal pathology [74–76]. However, this approach is often not well tolerated in older adults and frequently requires a feeding tube, which can be an additional financial burden on the patients. Systemic steroids are used for acute exacerbations while topical glucocorticoids are used to provide long-term control [121,122]. In addition to dietary manipulation some other treatment strategy such as steroid therapy is in use and anti-cytokine therapy (anti-IL-5 and anti-IL-4Rα) is on clinical trial. The drawback of anti-cytokine therapy is similar to the elimination diet treatment; the EoE symptoms come back as soon as therapy is withdrawn. Another potential pharmacological agent currently on trial is anti-IL-13 neutralizing antibody treatments for EoE. However, it is debatable whether an anti-IL-13 treatment strategy will be successful, as IL-13-induced EoE is dependent on IL-5 and IL-13 gene deficiency does not impair antigen-induced EoE [62]. A recent clinical trial of anti-IL-13 showed that esophageal eosinophilia is reduced only ~60% and the trail did not achieve primary endpoint following 6 months of treatment [123]. This clinical finding is in accordance with earlier reported studies that IL-13 is not critical in EoE pathogenesis [62]. The topical or systemic steroids treatment for EoE significantly reduces both clinical and histological symptoms. Swallowed steroid therapy was found to be more effective with the complete remission in a significant number of patients compared with dietary therapy. But, there are limitations that exist with oral swallowed steroids therapy: dependency on treatment (reoccurrence of EoE symptoms within few months after discontinuation of therapy). Our, most recent report indicates that IL-18 and iNKT cells are induced in EoE patients and IL-18 activate iNKT cells; therefore neutralization of iNKT cells, IL-18 or IL8Rα may be a possible therapeutic target for EoE [124,125]. Taken together, much progress has been made concerning the EGID therapy, but there is still lack of mechanistic understanding compared with other cell types and gastrointestinal diseases like IBD. We hope that in near future a better understanding of the pathogenesis and treatment of EGIDs will emerge.

## Conclusion

**Figure 6:**
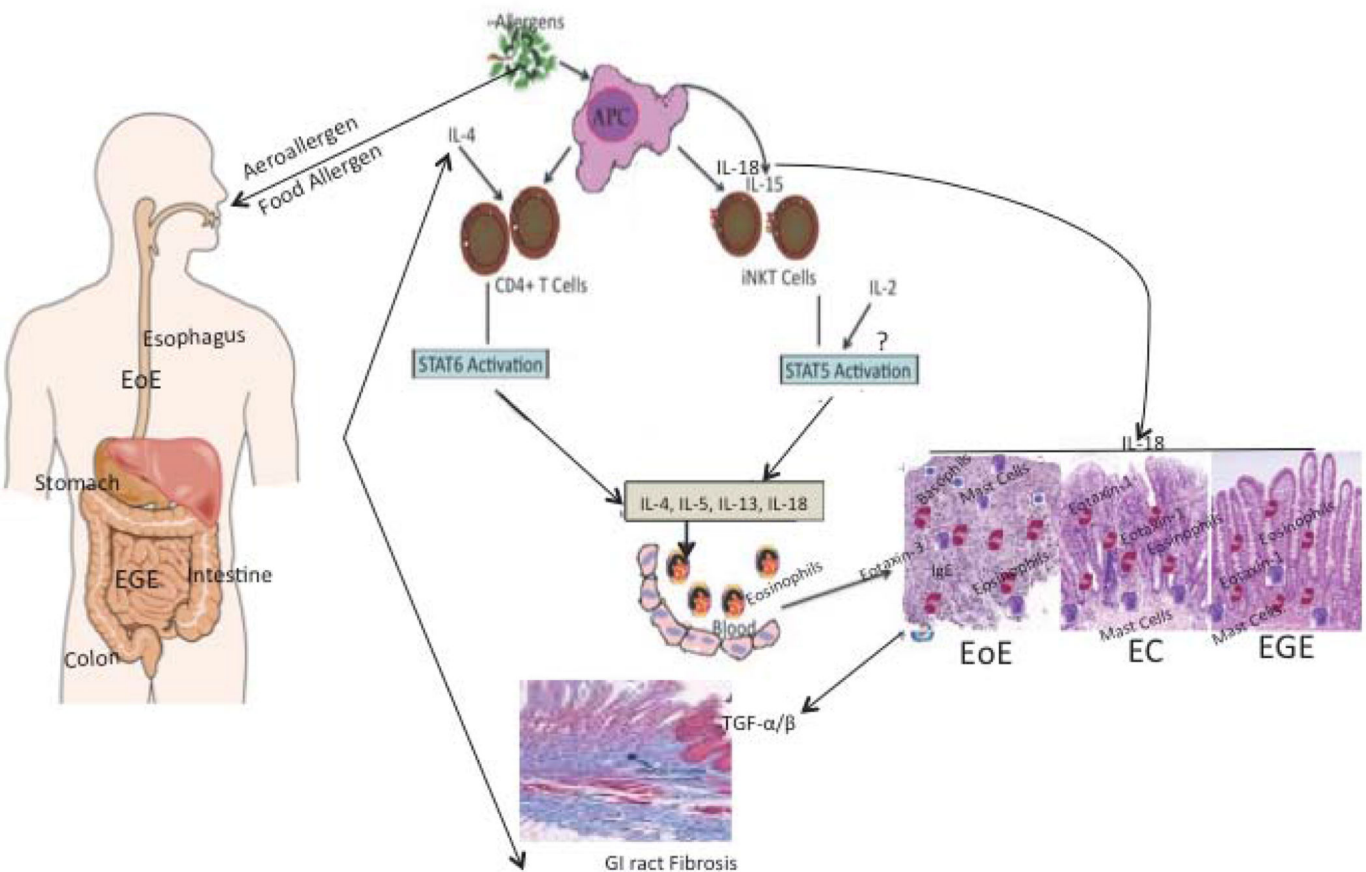
Diagrammatic representation of possible mechanistic pathway for the induction of EGID. Allergens (Aero- or food-) procured by antigen presenting cells (APCs) are offered to the conventional (CD4+ T cells) and non-conventional T cells (iNKT cells). Based on our earlier reports and presented information in the review, we propose that both conventional CD4+T cells and non-conventional iNKT cells generate eosinophil active Th2 cytokines like IL-4, IL-5, IL-13. The iNKT cells require IL-15 and IL-18 for survival, proliferation and activation. The antigen presenting cells may be the major source of IL-15 and IL-18 to activate iNKT cells to initiate the disease process in a number of gastrointestinal disorders like EoE, EGE and EC. IL- 18 is a unique cytokine that has a role in a number of allergic diseases and recent publication indicates that IL-18 overexpression in the tissues promotes eosinophilic inflammation and tissue remodeling through the induction of TGFα/β. Our earlier publications and evidences presented in this review indicate that STAT5 and STAT6 regulate the activation of CD4+ T and iNKT cells, respectively. Interestingly, the role of eosinophil active Th2 cytokines and chemokines (eotaxins-1, and eotaxin-3) in gastrointestinal epithelial mucosa is critical in the accumulation of eosinophils into the esophagus (via eotaxin-3 involvement) and in the intestine and colon (via Eotaxin-1 involvement), respectively, which promotes EGID.

We presented a detailed understanding on eosinophil associated gastrointestinal disorders in this review to understand the development of EGID pathogenesis (Figure 6). In brief, we have discussed the eosinophil biology and its association to the food allergy, specifically the role of CD4+ T cells and iNKT cells, in the EGID pathogenesis. We discussed the significance of food allergy including B-cell derived IgE in the EGID pathogenesis. EGID patients and murine models showed that these factors are critical for promoting EGID. Interestingly, FcεRI and FcεRII receptors on blood cells are differentially expressed in EGID compared with normal subjects. We have also provided the evidence for the involvement of eosinophils, mast cells and basophils in tissue remodeling during the development of EGID pathogenesis. Additionally, we have also discussed the major role of Th2 cytokines, IL-5 and IL-13, IL-15, IL-18 chemokines and eotaxins in the development of symptoms of EoE and EGE. Most recently, we identified additional key targets, IL-15, IL-18 and its responsive iNKT cells that play a critical role in initiation and progression of EoE, apart from IL-5, IL-13 and eotaxins. Earlier, clinical trial of anti-IL-5, and recent clinical trial with anti-IL-13 reported reduced esophageal eosinophilia, but both failed to achieve primary endpoint goals for EoE therapy [123]. Therefore, now the attention should be given on the source of these cytokines in EGID therapy. Data from overexpressing IL-15, IL-18-transgenic mice, IL-15Rα- deficient mice, iNKT cell-deficient mice, and depletion of iNKT cells via anti-CD1d or anti-Vα24/Jα18 antibodies demonstrate the critical role of IL-15, IL-18, CD4+ T cells, iNKT cells and their receptors in the development of characteristic features of EGID (Cartoon Figure 6 for proposed detailed mechanistic pathway). These data provided us with novel therapeutic targets for EGID treatment and diagnosis. Studies with human subjects confirmed their association with disease pathogenesis. Hence, the target molecules like iNKT cells, IL-18, anti-CD1d or anti-Vα24/Jα18 antibodies can be utilized for the future diagnosis and therapeutic treatment strategies for EGID.
